# Preliminary Exploration of Transpedal Lymphangiography With High-Dose Ethiodized Oil Application in the Treatment of Postoperative Chylothorax

**DOI:** 10.3389/fmed.2021.754781

**Published:** 2021-12-15

**Authors:** Lin Li, Xin Wu, Dehan Liu, Wei Zhang, Lian Yang, Feng Pan

**Affiliations:** ^1^Department of Radiology, Union Hospital, Tongji Medical College, Huazhong University of Science and Technology, Wuhan, China; ^2^Hubei Province Key Laboratory of Molecular Imaging, Wuhan, China

**Keywords:** lymphangiography, ethiodized oil, chylothorax, postoperative complications, thoracic neoplasms

## Abstract

**Objective:** To preliminarily explore the safety and effectiveness of transpedal lymphangiography (TL) with high-dose ethiodized oil application (>20 ml) in the treatment of high-output postoperative chylothorax.

**Methods:** From 1 July 2020 to 1 July 2021, a total of 7 patients with high-flow postoperative chylothorax (> 1,000 ml/d) were retrospectively reviewed in a single center. Clinical data, including surgery types, technical and treatment success of TL, and adverse events of TL, were collected and analyzed.

**Results:** Seven patients (5 cases of non-small cell lung cancer; 2 cases of esophageal carcinoma) with a median age of 62 years (range: 30–70 years) occurred postoperative chylothorax after tumor resection with mediastinal lymphadenectomy. All patients received conservative treatment including total parenteral nutrition and somatostatin administration for a median of 20 days (range: 15–31 days) that failed to cure the chylothorax, so TL was performed as a salvage. Before TL, the median daily chyle output was 1,500 ml/day (range: 1,100–2,000 ml/day). The technical success rate of TL was 100% (7/7), with the median volume of ethiodized oil of 27.6 ml (range: 21.2–30.0 ml) injected in TL. Ruptured thoracic duct was identified in 5 patients (5/7, 71%) in fluoroscopy and chest CT after TL. The treatment success rate of TL was 86% (6/7). In 6 patients, the thoracic drainage was removed after a median of 7 days (range: 4–13 days) from TL performance. No adverse event of TL was reported.

**Conclusion:** Transpedal lymphangiography with high-dose ethiodized oil application (>20 ml) is a feasible, safe, and effective modality for the treatment of high-flow (> 1,000 ml/day) postoperative chylothorax.

## Introduction

Postoperative chylothorax is a rare and refractory complication after thoracic surgery. It can lead to a large amount of nutrient loss, including protein, triglycerides, and other substances, resulting in electrolyte imbalance, malnutrition, and severe infection (1–3). Especially for patients with high-output chylothorax (> 1,000 ml/d), if treatment is ineffective, life-threatening sequelae might occur with a mortality rate of up to 50% (1–3). Conservative treatment (e.g., total parenteral nutrition, somatostatin, etc.) is the first-line choice for postoperative chylothorax (3–6). However, there are some defects when treating patients with high chyle output, such as unsatisfactory efficiency and long treatment period with poor tolerability (3–6). Surgical repair, as a salvage treatment after the failure of conservative treatment, has a high occurrence rate of secondary surgery-related complications and the risk of death (up to 38 and 25%, respectively) (4–7).

The conventional transpedal lymphangiography (TL) is a classic diagnosis method for postoperative chylothorax with a therapeutical effect. The viscous ethiodized oil injected in TL can embolize the lymphatic leakage site (8). In literature, TL was reported with an overall treatment success rate of over 50% and a complication rate of <3% when treating postoperative chylothorax (5, 7, 9). Compared to surgical repair, TL is minimally invasive and can be tolerated by critically ill patients. Therefore, TL holds the potential as second-line management where conservative treatment fails. In conventional TL, it was empirically recommended that ethiodized oil should not exceed 20 ml (5, 8–11). Nevertheless, this recommended ethiodized oil dose may be insufficient for treating high-dose postoperative chylothorax because of the poor treatment efficiency rate of 35% (5, 10). Increasing the ethiodized oil dose in TL may benefit the leakage embolization and improve treatment efficiency. So far, it still lacks objective evidence on the safety or clinical efficacy of TL treatment with high-dose ethiodized oil (> 20 ml) application in TL. This retrospective study preliminarily explored the safety and effectiveness of TL with high-dose ethiodized oil application (> 20 ml) in the treatment of high-output postoperative chylothorax when conservative treatment failed.

## Materials and Methods

### Definitions and Standards

**Figure 1:**
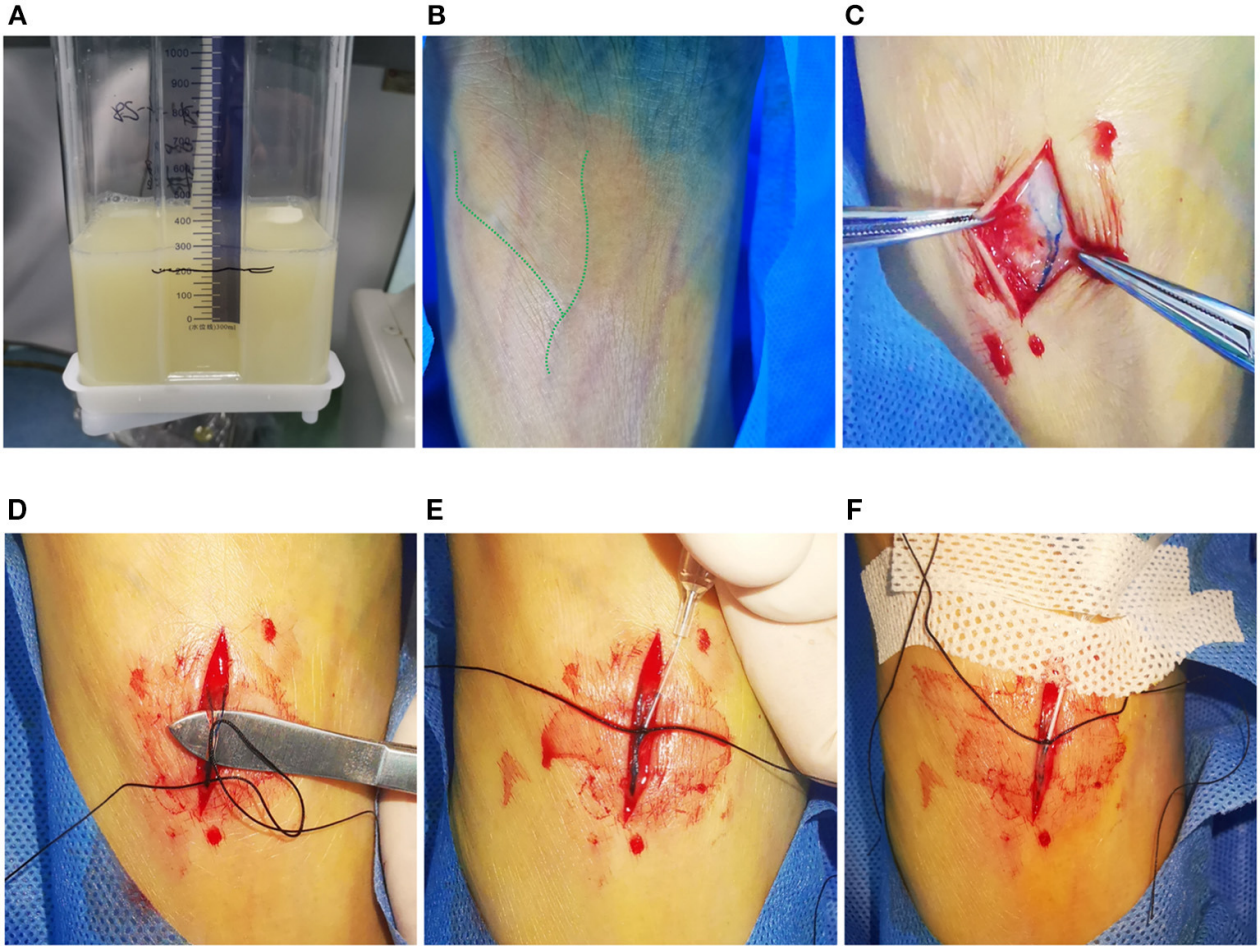
Typical visual demonstration of chylothorax drainage and cannulation of pedal lymphatic vessel in TL procedure. **(A)** Typical visual demonstration of chylothorax drainage. **(B)** 20 min after dye injection between interdigital spaces, superficial lymphatic vessels could be observed with tinted blue color (alongside the green dash line) on dorsal foot. **(C)** After incision, subcutaneous lymphatic vessels can be clearly found with deep blue color. **(D)** Meticulous separation of the target lymphatic vessels. **(E)** Manual puncture of the lymphatic vessel by using a 26-G intravenous needle. **(F)** After fixation of the trocar by suture and sterile tape, the ethiodized oil can be injected afterwards.

The clinical and pathological staging of malignant tumors is based on the 8th edition of TNM Classification of Malignant Tumors (12, 13). Diagnostic criteria for postoperative chylothorax: 1. Exclusion of other postoperative complications of pleural effusion, such as malignant pleural effusion, inflammatory exudation, etc.; 2. Milk-like pleural drainage (Figure 1A), triglyceride level of drainage fluid higher than 110 mg/dL, or positive chylomicrons test (11, 14, 15). The technical success of TL is defined as the opacification of the thoracic lymphatic system under fluoroscopy (11, 16, 17). The treatment success of TL is defined as immediate cessation or gradual reduction of lymphatic leakage after TL leading to the removal of thoracic drainage within 2 weeks without the requirement of other treatments (6, 11, 18, 19). The lymphatic duct leakage is identified as definite ethiodized oil extravasation out from thoracic duct visualized in dynamic fluoroscopy, or the ectopic presence of the ethiodized oil in the pleural space observed in chest CT after TL (20).

### Patients

This study retrospectively collected a total of 7 patients with postoperative chylothorax after thoracic surgery admitted to this center from July 2020 to July 2021. The TL was indicated following: 1. fulfill the postoperative chylothorax diagnostic criteria; 2. after conservative treatment including total parenteral nutrition and somatostatin for more than 2 weeks, no progressive decrease of the thoracic chyle output; 3. after multidisciplinary team discussion (including physicians from departments of respiratory medicine, thoracic surgery, interventional radiology, and anesthesiology), it was decided to give priority to TL treatment; 4. obtain written informed consent of the patient. Contraindications for TL treatment follow: severe pulmonary dysfunction identified by pulmonary function test, pulmonary arteriovenous malformations/fistula, and right-to-left shunt heart disease (11).

### TL Procedure

After dermal sterilization, 1 ml of a 1:3 mixture of methylthioninium chloride (20 mg/2 ml, JUMPCAN pharmaceutical group Co., LTD, Jizhou, China) and 1% lidocaine was injected into each interdigital space on either left or right foot to dye the dorsal lymphatic vessels (Figure 1B). After 20 min, incise the dorsal skin of the foot under local anesthesia to separate the subcutaneous lymphatic vessel (Figures 1C,D). Afterwards, puncture the target lymphatic vessel by using a 26-G trocar needle (Jerui, WEGO, Weihai) (Figure 1E) and fix it with suture and sterile tapes (Figure 1F). Then, connect the trocar with an infusion pump (WZS-50F6, Zhejiang Smiths Medical, Hangzhou, China) to conduct the ethiodized oil injection (super-fluid ethiodized oil, Hengrui, Jiangsu, China). The pressure restriction and infusion velocity of the pump was set as a “High” model and 0.4 ml/min, respectively. From then, fluoroscopy (Artis Zee, Siemens Healthineers, Erlangen, Germany) was performed every 2–5 min to dynamically observe the opacification of the lymphatic vessels from the foot to the left jugular vein angle. Unlike the previous reports in which the operator ceased the injection after 6 to 12 ml of ethiodized oil application no matter whether the thoracic duct was opacified, the endpoint of injection in this study was the visualization of the left jugular vein angle (20–22). Then, the needle was removed and the dorsal wound of the foot was sutured. The chest CT (SOMATON emotion 16, Siemens, Germany) was immediately performed (filling phase) to identify the specific lymphatic leakage site with 3-dimensional reconstruction (scan parameters: 120 kv, adaptive current, and B30f iterative reconstruction) (20). If there is no lymphatic fistula, the same chest CT examination will be re-performed 24 h later (nodal phase) for further diagnosis (20).

### Study Goals

Record the time of the lymphatic opacification at the levels of the knee joint space, upper femoral head, L5/Th12/Th5 vertebrae, and left jugular veinous angle from start of ethiodized oil injection during TL. Identify the technical success of TL and thoracic duct rupture in fluoroscopy and following chest CT. Evaluate the treatment success and adverse event of TL from the institutional electronic records.

### Statistical Analysis

The data in this study were analyzed using Excel 2019 (Microsoft, USA). Quantitative and counting data are presented as median with range and count with percentages of the total, respectively. Since no group comparison is performed, only descriptive analysis is used.

## Results

The median age of 7 patients (4 males and 3 females) was 62 years (range: 30–70 years). Two and 5 patients were diagnosed with esophageal carcinoma and non-small cell lung carcinoma, respectively. All patients underwent surgical tumor resection with mediastinal lymphadenectomy. After surgery, postoperative chylothorax occurred in 5 patients on the right side and 2 on the left side. TL treatment was performed at a median of 20 days (range: 15–31 days) after surgery. The median daily chyle output before TL was 1,500 ml/day (range: 1,100–2,000 ml/day). The details are shown in Table 1.

**Table 1:** Basic characteristics.

**Patient ID**	**Sex**	**Age (years)**	**Diagnosis**	**Clinical staging**	**Surgery**	**Mediastinal lymphadenectomy**	**Location of the tumor**	**Postoperative pathological staging**	**Pathological diagnosis**	**Side of chylothorax**	**Daily chyle output before TL (ml/d)**
1	Female	62	Non-small cell lung cancer	IA2 (1bN0M0)	Anatomical pulmonary resection	Yes	Upper lobe of right lung	IA2 (1bN0M0)	Invasive adenocarcinoma	Right	1,500
2	Male	64	Non-small cell lung cancer	IA2 (1bN0M0)	Anatomical pulmonary resection	Yes	Upper lobe of right lung	IA2 (1bN0M0)	Invasive adenocarcinoma	Right	1,100
3	Female	30	Non-small cell lung cancer	IA2 (1bN0M0)	Anatomical pulmonary resection	Yes	Lower lobe of right lung	IA2 (1bN0M0)	Invasive adenocarcinoma	Right	2,000
4	Male	62	Non-small cell lung cancer	IA2 (1bN0M0)	Anatomical pulmonary resection	Yes	Upper lobe of left lung	IA2 (1bN0M0)	Invasive adenocarcinoma	Right	1,500
5	Female	70	Non-small cell lung cancer	IA2 (1bN0M0)	Sublobar resection	Yes	Upper lobe of right lung	IA2 (1bN0M0)	Invasive adenocarcinoma	Left	1,200
6	Male	64	Esophageal carcinoma	II (T2N0M0)	Esophagectomy	Yes	Middle third of esophagus	IB (T2N0M0G1)	Well-differentiated squamous cell carcinoma	Left	1,700
7	Male	56	Esophageal carcinoma	II (T2N0M0)	Esophagectomy	Yes	Middle third of esophagus	IIA (T2N0M0G2)	Moderately differentiated squamous cell carcinoma	Right	2,000
Median (minimum-maximum)		62 (30–70)									1,500 (1,100–2,000)

*The clinical and pathological staging are based on the 8th edition of TNM Classification of Malignant Tumors (12, 13). TL, transpedal lymphangiography*.

**Figure 2:**
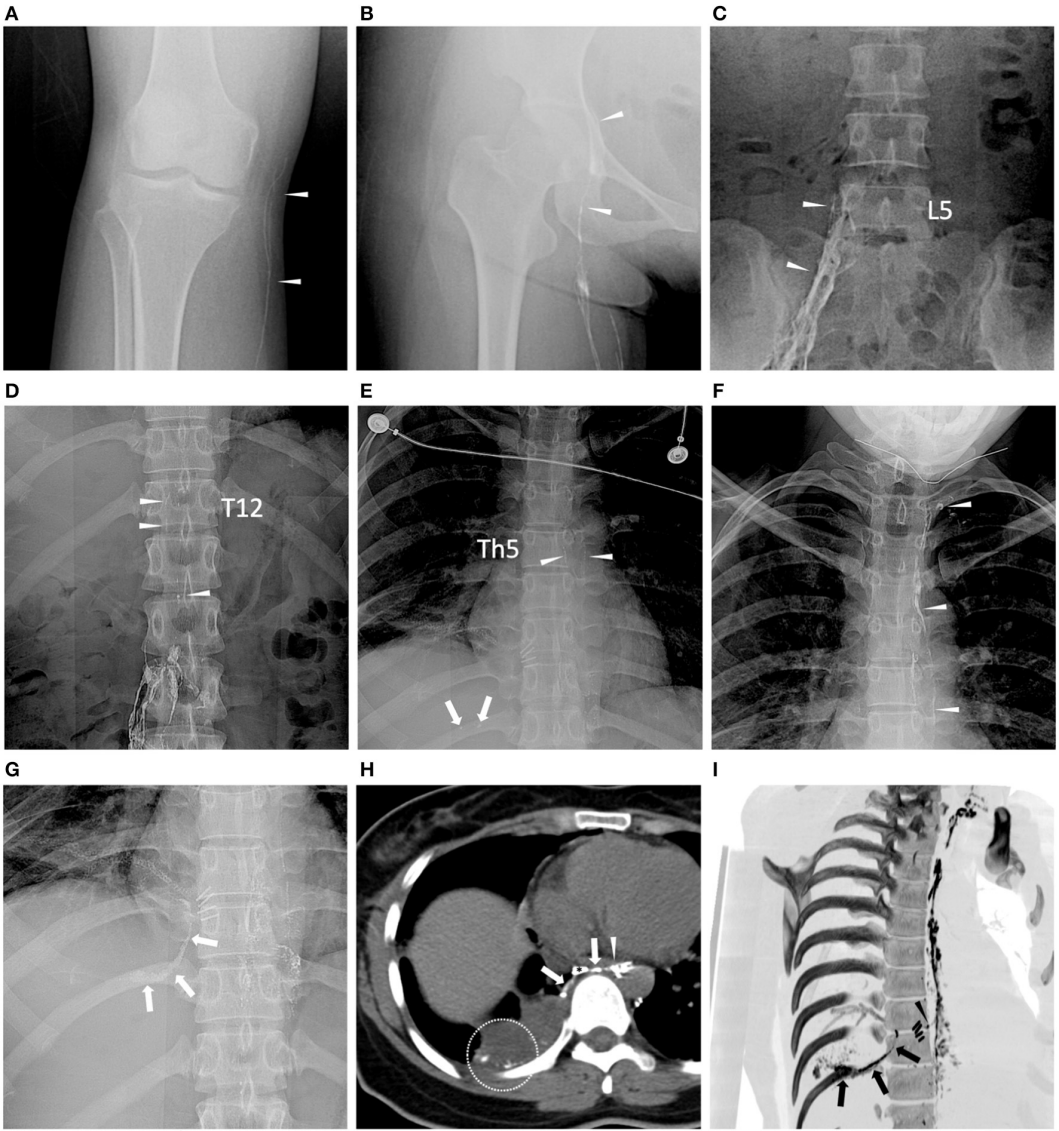
Dynamic opacification of lymphatic vessels in fluoroscopy and chest CT. Images were obtained from the same patient with non-small cell lung carcinoma (*Patient ID: 3*). **(A–F)** 3, 9, 22, 45, 52, and 60 min after ethiodized oil injection, respectively, lymphatic vessels (white arrowheads) at the level of knee joint space, upper femoral head, L5, Th12 and Th5 vertebrae, and left jugular venous angle were consecutively opacified and dotted ethiodized oil extravasation [**(E)** white arrows) can be observed in the course. **(G)** After completing the ethiodized oil injection (28.0 ml), fluoroscopy demonstrated more definite ethiodized oil extravasation (white arrows). **(H)** Following CT (axial image with 5 mm thickness) after TL showed a rupture of the thoracic duct (white arrowhead) near the metal clips (*) and thread-like ethiodized oil extravasation (white arrows) can be observed, as well as the free ethiodized oil in the right pleural effusion (within the white circle). **(I)** Reconstructed oblique CT image with maximum Intensity projection (MIP) showed more intuitive opacification of the thoracic duct, leakage site (black arrowhead) near the metal clips, and the ethiodized oil extravasation in the right pleural cavity (black arrows). It indicated a possibility of iatrogenic damage to the thoracic duct in the surgery.

The technical success of TL was 100% (7/7). The median volume of ethiodized oil injected in TL was 27.6 ml (range: 21.2–30.0 ml) (Table 2). The median time of lymphatic opacification at the levels of knee joint space, upper femoral head, L5 vertebra, Th12 vertebra, Th5 vertebra, and the left jugular venous angle from the start of ethiodized oil injection were 4 min (range: 3–7 min) and 10 min (range: 9–12 min), 22 min (range: 15–34 min), 42 min (range: 33–51 min), 52 min (range: 47–62 min) and 69 min (53–75 min) (Table 2; Figure 2). Lymphatic duct leakage, including definite ethiodized oil extravasation out from thoracic duct in dynamic fluoroscopy and the ectopic presence of the ethiodized oil in the pleural space in chest CT after TL, was identified in 5 patients (5/7, 71%) (Table 2; Figure 2). No Adverse event of TL was recorded.

**Table 2:** Details of TL treatment.

**Patient ID**	**Time of TL after surgery (days)**	**Foot of lymphatic cannulation**	**Technical success of TL**	**Time of lymphatic opacification level from ethiodized oil injection (min)**						**Volume of ethiodized oil injected in TL (ml)**	**Definite ethiodized oil extravasation identified in fluoroscopy**	**Ectopic presence of the ethiodized oil in the pleural space identified in filling-phase CT**	**Ectopic presence of the ethiodized oil in the pleural space identified in nodal-phase CT**	**Treatment success**	**Time of drainage removal after TL (days)**
				**Knee joint space**	**Superior margin of femoral head**	**L5**	**Th12**	**Th5**	**Left jugular venous angle**						
1	20	Right foot	Yes	5	10	15	40	60	75	30.0	Yes	Yes	n.a.	Yes	7
2	31	Left foot	Yes	7	10	30	50	55	69	27.6	No	No	No	Yes	13
3	16	Right foot	Yes	3	9	22	45	52	70	28.0	Yes	Yes	n.a.	Yes	8
4	15	Right foot	Yes	3	10	18	33	48	55	22.0	No	No	No	Yes	7
5	16	Right foot	Yes	3	12	22	42	47	53	21.2	Yes	Yes	n.a.	Yes	4
6	20	Left foot	Yes	4	10	24	39	50	63	25.2	Yes	Yes	n.a.	Yes	5
7	22	Right foot	Yes	5	11	34	51	62	72	28.8	Yes	Yes	n.a.	No	n.a.
Median (minimum-maximum)	20 (15–31)			4 (3–7)	10 (9–12)	22 (15–34)	42 (33–51)	52 (47–62)	69 (53–75)	27.6 (21.2–30)					7 (4–13)

*Filling-phase CT indicated simultaneous chest CT after TL, while nodal-phase CT indicated chest CT 24 h after TL (20). TL, transpedal lymphangiography; n.a., no applicable*.

After TL, 6 patients experienced a progressive decrease of the daily chyle output. The median time for removing the thoracic drainage after TL was 7 days (range: 4–13 days). The treatment success rate of TL was 86% (6/7). One patient without significant reduction of daily chyle output after TL underwent further percutaneous afferent lymphatic vessel sclerotherapy (ALVS) on the 8th day after TL, who was cured later (11).

## Discussion

In this retrospective study, 5 and 2 patients had chylothorax complications after lung cancer and esophageal cancer resection, respectively, with a daily chyle output of more than 1,000 ml/d. After 15–31 days of ineffective conservative treatment, all patients received TL treatment with high-dose ethiodized oil application (>20 ml). The technical success was achieved in all patients, with the median volume of ethiodized oil injected in TL of 27.6 ml. The treatment success rate of TL was 86% (6/7). No Adverse event of TL was reported.

So far, TL has been increasingly used to manage postoperative chylothorax with a technical success rate of 95–100% (5, 6, 8, 9, 23). Except for the embolization effect, ethiodized oil can induce sterile inflammation at the fistula, beneficial for subsequent histological repair (8). However, the traditional manual injection of ethiodized oil in TL was unstable with the velocity between 0.2 and 0.5 ml/min and tiring over hours' operation (8, 21). So, we improved the injection method by connecting an infusion pump commonly used, replacing the primordial handy injection. This simple technical improvement can stabilize the injection pressure and velocity. Moreover, it benefits the standardization of TL procedure for the research purpose. For instance, the mean time of lymphatic visualization at different targets can be statistically analyzed: 10 min for superior margin of femoral head (groin region), 42 min for Th12 (cistern chyli region), and 69 min for left jugular venous angle (whole thoracic duct).

In previous studies, the ethiodized oil usage in TL was mostly around 10 ml (5, 8–11, 18, 20–22). But after accomplishment of injection, the ethiodized oil probably only reached pelvic or abdominal lymphatic vessels, so interventionalists always needed to monitor for more than 4 h until the ethiodized oil flowed into the thoracic duct (8, 11, 20–22). With the increase of ethiodized oil dose to more than 20 ml, we can visualize the left jugular vein angle as the endpoint of ethiodized oil injection, resulting in a more reliable thoracic duct embolization. It explained why the treatment success rate of TL for the high-flow postoperative chylothorax reached 86% (6/7) in our cohort, which was higher than 35% in previous reports (5, 10). Similarly, a recent report also showed that high-dose ethiodized oil (>40 ml) application in intranodal lymphangiography (INL) was feasible and safe (24). It achieved a treatment success rate of 83% without any adverse event for the treatment of high-flow postoperative chylothorax, similar to our findings (24). However, we need to point out: in INL, part of the ethiodized oil was wasted because of the extravasation from the punctured lymph node, so we can't confirm how much of the ethiodized oil genuinely entered the lymphatic system (8, 24). But in TL, the needle was fixed with suture resulting in a more reliable ethiodized oil injection without any waste, resulting in less usage of ethiodized oil than INL (21).

Nevertheless, the interventionalists need to pay attention to the risk of ectopic embolism caused by ethiodized oil. Although most ethiodized oil in TL is filtered and gradually resolved in lymph nodes, a small portion of ethiodized oil in the lymphatic vessels can enter the subclavian vein into the pulmonary circulation, which was filtered by a regular pulmonary capillary bed (25). But in patients with comorbidities, such as severe pulmonary dysfunction, pulmonary arteriovenous malformations, and right-to-left shunt heart disease, the ethiodized oil might enter systematic circulation leading to lethal cerebral embolism (11, 25, 26). So, strict clinical estimation before TL is essential.

Among the 7 patients, the lymphatic duct leakage presenting with definite ethiodized oil extravasation out from thoracic duct and the ectopic presence of the ethiodized oil in the pleural space was identified in 71% of patients (5/7), similar to 64–86% reported in the literature (10, 14, 21, 27). It implied an iatrogenic injury of the thoracic duct in the prior surgeries. Chest CT after TL is routinely performed in our cohort because it has higher diagnostic sensitivity for leakage site identification than fluoroscopy. It can provide more explicitly anatomical details of the lymphatic system (20, 28, 29). If TL treatment fails, these CT data can also assist in planning further lymphatic interventional therapies, such as ALVS mentioned above and thoracic duct embolization (8, 9, 11, 20, 22).

This study has limitations. First, the sample size is minimal. Second, it lacks a comparison to conventional TL with ethiodized oil usage of <20 ml. In the cases with high-dose postoperative chylothorax (>1,000 ml/d), conventional TL showed a poor efficiency rate of about 35%, so it was rarely solo performed (5, 10). Instead, conventional TL with percutaneous lymphatic intervention (e.g., thoracic duct embolization or ALVS) was the optimal choice with a higher efficiency rate of 75–100% (9). However, by comparing the literature, the results of our study suggest that TL with high-dose ethiodized oil application probably has a better therapeutic effect.

In summary, the results in this preliminary study suggest TL with high-dose ethiodized oil is a feasible, safe, and effective method for treating high-flow postoperative chylothorax. It holds the potential as the second-line choice when conservative treatment fails. Besides, this study works out stable ethiodized oil injection using a commonly used infusion pump, advantaging TL performance.

## Data Availability Statement

The raw data supporting the conclusions of this article will be made available by the authors, without undue reservation.

## Ethics Statement

The studies involving human participants were reviewed and approved by Ethics Committee of Union Hospital, Tongji Medical College, Huazhong University of Science and Technology. The patients/participants provided their written informed consent to participate in this study.

## Author Contributions

FP, LL, and XW contributed to the conception, design of the study, and wrote sections of the manuscript. WZ, DL, and LY collected, analyzed, and interpreted the data. All authors contributed to the article and approved the submitted version.

## Conflict of Interest

The authors declare that the research was conducted in the absence of any commercial or financial relationships that could be construed as a potential conflict of interest.

## Publisher's Note

All claims expressed in this article are solely those of the authors and do not necessarily represent those of their affiliated organizations, or those of the publisher, the editors and the reviewers. Any product that may be evaluated in this article, or claim that may be made by its manufacturer, is not guaranteed or endorsed by the publisher.
