# Corrigendum: Preliminary Exploration of Transpedal Lymphangiography With High-Dose Ethiodized Oil Application in the Treatment of Postoperative Chylothorax

**DOI:** 10.3389/fmed.2022.929540

**Published:** 2022-05-19

**Authors:** Lin Li, Xin Wu, Dehan Liu, Wei Zhang, Lian Yang, Feng Pan

**Affiliations:** ^1^Department of Radiology, Union Hospital, Tongji Medical College, Huazhong University of Science and Technology, Wuhan, China; ^2^Hubei Province Key Laboratory of Molecular Imaging, Wuhan, China

**Keywords:** lymphangiography, ethiodized oil, chylothorax, postoperative complications, thoracic neoplasms

In the published article, there was an error in **affiliation 1**. Instead of “Department of Radiology, Tongji Medical College, Union Hospital, Huazhong University of Science and Technology, Wuhan, China”, it should be “Department of Radiology, Union Hospital, Tongji Medical College, Huazhong University of Science and Technology, Wuhan, China”.

The authors apologize for this error and state that this does not change the scientific conclusions of the article in any way. The original article has been updated.

## Publisher's Note

All claims expressed in this article are solely those of the authors and do not necessarily represent those of their affiliated organizations, or those of the publisher, the editors and the reviewers. Any product that may be evaluated in this article, or claim that may be made by its manufacturer, is not guaranteed or endorsed by the publisher.

